# Foreign Correspondence

**Published:** 1889-04

**Authors:** 


### Foreign Correspondence.

To the Editor:

In a confidential letter shown me by my friend, Dr. I. B. Davenport, Dr. Thackston, although speaking in the kindest manner about the French Dental Congress of 1889, expressed the fear that this congress might injure the Dental Section of the General International Medical Congress, whose next session is to be held in Berlin in 1890.

Dr. Thackston fears that the organizers of this French congress would be mostly all schismatics, who abstain from general international reunions, and who would not hesitate to raise up altar against altar. I can boldly affirm that this idea has never entered the mind of any one among us in France. The Paris Exposition in 1889 will certainly attract a large number of our colleagues from all lands. Why should we not take advantage of the occasion to discuss subjects interesting to all of us, questions of practice, professional organization, etc.? While acting upon this idea we had no thought of hostility towards the International Medical Congress of 1890.

The ophthalmologists have held their seventh international congress, the last session taking place at Heidelberg in 1888, if I am not mistaken. The principal organizers of this congress regularly follow the sessions of the International Congress, and it never occurs to them that a reunion of specialists can interfere in any way with a general reunion. There will also be held in Paris in 1889 a Hygienic Congress. Those who take part in it certainly have no thought prejudicial to the hygienic section of the general congress. Why then should intentions be attributed to dentists which no one would attribute to oculists or hygienists? It seems to me that an international dental congress can be held with perfect propriety without in the least interfering with the general international medical congress. Very respectfully yours,

George P. E. Kuhn.

3 Rue Scribe, Paris, Jan., 1889.

---

To the Editor:

Implantation is still all the rage here, and all kinds of experiments are being tried. Wishing to know if the periosteum really was necessary on the roots of teeth, and really was a factor in success, I scraped and polished the roots of two lateral incisors for implantation and held them firmly for two weeks each; but at the end of that time they slipped out of their own accord on the removal of the retaining plate; in one case there was some necrosis of the socket, but the other socket was perfectly healthy and the gum normal. Patients each 40 years of age.

I am very much interested in the trials of the implantation of porcelain teeth with porcelain roots, which were mentioned in some recent society meetings. Can you give any information as to the results?

The great difficulty here is to get suitable teeth, and I was very much surprised to learn from a Swiss-American colleague, Dr. Wetzel, of Basel, who allows me to use his name, that he gets the best teeth and has success in implanting them from the subjects in the dissecting-room. This was news to me, but he says that it has been the practice with several whom he knows. Teeth used to be gathered on European battle fields, and sold in sets by supply dealers for artificial dentures in old times before porcelain teeth were made, so there is nothing new under the sun except the Low Patents,

These patents do not trouble us in Switzerland, however; for no patent laws have existed in this country of ingenious people. and they have had free use of the inventions of all nations, and the new laws of last year only allow patents for inventions made after the passage of the patent laws. With the use of the world's inventions it was found that the Swiss, though naturally most clever and inventive and the first successful makers of watches, were falling behind those countries which had patent laws, and only regained their waning prestige in watch-making after seeing and buying the improved machinery exhibited at the Philadelphia exposition. The authorities find that inventive genius needs stimulation and protection. The professional (?) movement in America to discourage dental patents seem most short-sighted considering the fact that the grand achievements of American dentistry have been gained with the splendid and unequalled instruments patented by American practitioners. There are glaring abuses and serious faults in the system which need correction and reform, but if the profession expect the march of improvement and invention to keep pace with their daily increasing and labor-saving machinery, they will have to grant the necessary reward to stimulate the tired practitioner, to whom an idea of a new investment has occurred during his day's labor, to give up his hours of needed recreation and rob himself of his well-earned rest, to make and perfect and put on the market that which will assist his fellows and be a boon to mankind.

Renewing my thanks for the Topical Index I am,

Yours fraternally,

Basel, Feburary 4, 1889. L. C. BRYAN.

To the Editor:

Among the various reforms that have taken place here, during the last ten years, one of the most important is now being introduced, viz.: establishing a dental school at the regular medical school. Guy's Hospital Medical College, one of the largest and oldest in the kingdom, has undertaken the experiment for many years. Guy's Hospital has had a dental department. F. Newland Pedley, F.R.C.S., has during his connection with this department still further developed its utility. Lately he has contributed some very valuable and forcible articles upon "Dental Reform in General Hospitals," and upon Dental Scools at Medical Colleges. At a recent meeting of the Governor's of Guy's Hospital, it was decided to erect suitable buildings to carry out this scheme. Opinion is divided upon this innovation, feeling so proud and grateful for what the special schools and hospitals have done, we shall watch with interest this new school, the success that has been attained in America, of the Dental Departments of Universities may throw some light upon the future of "Medico-Dental Schools.

Brighton, Eng. Walter Harrison.

---

To the Editor.—The British Dental Association will meet in August, at Brighton ("the Queen of the watering places"), this year. President Samuel Lee Rymer, J.P.L.D.S. (Croyden) and the local Secretary, J. H. Redman, D.D.S. Dr. W. G. A. Bonwill has accepted an invitation to attend the meeting of the British Dental Association at Brighton the coming summer, and clinic on his special methods of practice. He will also attend the Dental meetings in London, Berlin, and the International Congress, in Paris. XX.

---

### CHANGE IN NAME.

The Welch Dental Company has been merged into the Wilmington Dental Manufacturing Company with largely increased capital. No change will be made in the membership of the new firm; they, virtually, being the same, although heretofore doing business under two separate names.

